# A story of (in)coherence: climate adaptation for health in South African policies

**DOI:** 10.1093/heapol/czae011

**Published:** 2024-02-28

**Authors:** Amanda V Quintana, Susannah H Mayhew, Sari Kovats, Lucy Gilson

**Affiliations:** Faculty of Public Health Policy, London School of Hygiene and Tropical Medicine, Keppel Street, London WC1E 7HT, United Kingdom; Faculty of Public Health Policy, London School of Hygiene and Tropical Medicine, Keppel Street, London WC1E 7HT, United Kingdom; Faculty of Public Health Policy, London School of Hygiene and Tropical Medicine, Keppel Street, London WC1E 7HT, United Kingdom; Faculty of Public Health Policy, London School of Hygiene and Tropical Medicine, Keppel Street, London WC1E 7HT, United Kingdom; Division of Health Policy and Systems, School of Public Health and Family Medicine, University of Cape Town, Observatory, Cape Town 7925, South Africa

**Keywords:** Climate change, health policy, policy analysis

## Abstract

Climate adaptation strengthens and builds the resilience of health systems to future climate-related shocks. Adaptation strategies and policies are necessary tools for governments to address the long-term impacts of climate change and enable the health system to respond to current impacts such as extreme weather events. Since 2011 South Africa has national climate change policies and adaptation strategies, yet there is uncertainty about: how these policies and plans are executed; the extent to which health policies include adaptation; and the extent of policy coherence across sectors and governance levels. A policy document analysis was conducted to examine how South African climate change, development and health policy documents reflect the health adaptation response across national and Western Cape levels and to assess the extent of coherence across key health and environment sector policy documents, including elements to respond to health-related climate risks, that can support implementation. Our findings show that overall there is incoherence in South African climate adaptation within health policy documents. Although health adaptation measures are somewhat coherent in national level policies, there is limited coherence within Western Cape provincial level documents and limited discussion on climate adaptation, especially for health. Policies reflect formal decisions and should guide decision-makers and resourcing, and sectoral policies should move beyond mere acknowledgement of adaptation responses to a tailored plan of actions that are institutionalized and location and sector specific. Activities beyond documents also impact the coherence and implementation of climate adaptation for health in South Africa. Clear climate risk-specific documents for the health sector would provide a stronger plan to support the implementation of health adaptation and contribute to building health system’s resilience.

Key messages

- Climate adaptation strategies and policies are necessary tools for governments to address the projected and long-term impacts of climate change and provide an opportunity to strengthen and build the resilience of health systems to future climate-related shocks.
- While South African health sector documents largely commit to mainstreaming climate change adaptation, they do not include specific elements that are critical to support implementation.
- The findings show that there is an overall incoherence in South African climate adaptation for health policies at both the national and Western Cape provincial level.
- As a cross-cutting issue, climate change should move beyond mere acknowledgement in sectoral plans and a tailored response plan of actions that are institutionalized and geographic and sector specific should instead be presented.

## Introduction

It is no longer contested that climate change directly and indirectly impacts health outcomes. Climate adaptation, ‘the process of adjustment to actual or expected climate and its effects’ (Noble *et al*., 2014), presents an opportunity for the health sector to strengthen and build the resilience of the health system to future climate-related shocks and impacts. South Africa is vulnerable to climate change and extreme weather impacts, as seen in the 2017/18 Drought in Western Cape and devastating floods in KwaZulu-Natal in 2022.

In 2011, the hosting of the COP17 meeting in Durban, South Africa led to the development of the National Climate Change Response Policy (NCCRP) that supported the inclusion of climate change adaptation into policies and strategies across all tiers of government (Myers and Rother, 2012). The NCCRP was the first climate change policy document for South Africa that identified the health sector as a priority adaptation sector. Climate change adaptation policies are tools available to governments, or a group of actors, that are designed to address the projected and long-term impacts of climate change (Dupuis and Biesbroek, 2013). They include policies or plans that support adaptation even when not labelled as such, for example, activities to protect the population from disasters and climate risks such as heat or droughts. Adaptation plans and actions therefore allow a system, such as the health system, to respond to the current impacts of climate change and ameliorate future impacts. In South Africa, there are a plethora of climate change policies and adaptation strategies, yet there is a growing uncertainty about how policies and plans for climate change adaptation in the health sector are executed at the local and national level (Chersich and Wright, 2019). Thus, assessing the coherence among related national and subnational policies offers value by revealing the ways in which health adaptation is addressed in South Africa, as well as in considering the potential for implementing such strategies in the health sector (Darjee *et al*., 2021).

### Policy coherence

The importance of mainstreaming climate change adaptation strategies across governance levels and sectors is well documented (Urwin and Jordan, 2008; Stringer *et al*., 2014; England *et al*., 2018). Mainstreaming of key issues ensures policy-makers consider particular perspectives in creating a policy. Some scholars suggest that policies that are aligned can help to increase the effectiveness of adaptation by improving coordination and processes for improved outcomes (UNFCCC, 2017; Bouyé and Walther, 2018). Policy coherence can be understood as a logical consistency across all dimensions of policy, including sectors, across governance levels and for implementation (Nilsson *et al*., 2012). Further, it refers to relationships between policies whose objectives influence one another, as a sectoral policy can be effective in achieving its objectives without being coherent in relation to other policy areas. Others see coherence as critical to ‘good governance’, especially in climate governance where climate change is a cross-cutting challenge and is fragmented across sectors and governance levels within a country (Jordan and Lenschow, 2010).

To date, literature looking at the policy coherence of climate adaptation has considered non-health sectors. Ranabhat *et al.* (2018) looked at the forestry sector in Nepal and found coherence in the motivation and adaptation measures but not on implementation; Zembe *et al.* (2022) found incoherencies among disaster risk reduction, climate adaptation and food security policies in South Africa; and finally, England *et al.* (2018) looked at climate change adaptation in water, agriculture, forestry and energy sectors in sub-Saharan African countries and uncovered differing degrees of coherence where actions to address immediate challenges were more coherent than longer-term climate strategies. As no literature has looked at the policy coherence between policies on climate change in or with the health sector, this paper aims to address this gap.

South Africa has a devolved governance system where subnational—provincial—governments are semi-autonomous. In such systems, there is a possibility of divergence in focus between national and subnational level policies addressing the same issue or between a policy’s original purpose and its translation at subnational level. South Africa has climate change policies that include sector-specific climate response plans; however, the extent to which health policies include climate adaptation strategies or considerations varies (Dos Santos *et al*., 2019; Godsmark *et al*., 2019).

This paper analyses existing policy documents relevant to climate change adaptation for health at the national and one provincial government level in South Africa to (1) depict how adaptation is considered and understood in documents at each governance level and (2) assess the coherence or incoherence across key health and environment sector documents that include elements decision-makers engage with to respond to health-related climate risks and support proper implementation.

## Methods

Taking a systems thinking approach and conducting thematic and inductive analysis, documents were reviewed across the health, environment and development sectors at the national and Western Cape provincial level in order to capture information on climate adaptation for health in the country. The Western Cape province was chosen as the province of focus because of its experience of drought, a possible indicator of climate change, and because of the expertise in climate change policy present within the province.

The Dalglish *et al.* (2020) systematic approach to policy document review READ approach was utilized to first select and read documents for analysis, secondly extract important data into a comprehensive spreadsheet, thirdly analyse the information extracted and finally distil the findings (Dalglish *et al*., 2020).

### Selection of documents and content analysis

Strategy and planning documents contain written and formal statements of intent by decision-makers that can bring an understanding of policy commitments and plans for action. National and Western Cape province policy documents were selected for examination for the period 2011, when the first National Climate Change Response White Paper (NCCRP) was released in March 2022. A range of legal and official documents as well as working documents, such as white papers and reports, were selected, which focused on climate change adaptation, health, development and disaster risk management. Any additional policy documents that address one of two climate risks with direct and indirect impacts on health in the Western Cape were included (heat and drought). Selected health documents were expected to include climate change adaptation information for health actors and were not focused on the determinants of health. South African government webpages and general web-engine searches helped to identify documents. Additional documents were included if referred to in a reviewed document or identified by a key stakeholder. The most recent version of each document was reviewed, except in cases where another document referred to a previous version; for example, the previous iteration of the National Health Strategic Plan was referred to by the 2011 NCCRP.

Of the 33 documents initially selected, 6 were excluded as a more recent version of the same document existed, resulting in a final total of 27 documents examined. During the final step of the READ approach, a series of analyses were conducted.

A content analysis was undertaken of 16 national and 11 Western Cape provincial level documents (summarized in Supplementary Annexe 1). This summative content analysis sought to understand the contextual use of words and content (Potter and Levine-Donnerstein, 1999) to identify patterns, themes or intents (Hall and Steiner, 2020) around discussions and definitions of adaptation, specifically health adaptation. This content analysis helped to assess information on health adaptation available to decision-makers when working to implement policy.

### Policy coherence analysis

The policy coherence analyses entailed cross-reference mapping, assessment of measures of health adaptation and implementation process analysis. Cross-reference maps were developed to depict inter- and cross-sectoral references at each governance level, using the initial 33 selected documents. During the READ data extraction phase, note was taken of when each document referenced other relevant documents. These references included whether the document expressed alignment with another document, an acknowledgement of another document or information from another document. Each document’s institutional author was captured to organize documents by sector—environment, health or development. These referencing connections were then mapped visually to depict the extent of intra-sector and cross-sector referencing at each governance level.

The next step of the policy coherence analysis focused on key documents that had cross-sector references, identified in the maps or included health adaptation content, determined from the content analysis. There were six of these documents in total: four at the national and two at the Western Cape provincial level. Of these six documents, three documents were authored by the health sector and three by the environment sector.

Finally, a modified policy coherence for development framework, as used in Ranabhat *et al.* (2018), was utilized to assess policy elements identified as important to support implementation (Ranabhat *et al*., 2018). The elements considered were policy instruments and implementation practices (Nilsson *et al*., 2012; Harahap *et al*., 2017; Ranabhat *et al*., 2018). Policy instruments are assessed under the ‘measures for health adaptation analysis’ and implementation practices are analysed under the ‘implementation process analysis’. Coherence or incoherence of measures can highlight areas of potential synergies or conflict among policy documents, while implementation elements can show, for example, whether institutional actors or stakeholders are given complementary or competing roles to address health adaptation.

To better understand the extent to which actions that reduce climate change-related risks to health in South Africa, the ‘measures for health adaptation analysis’, looked for the existence of measures related to adaptations for health across the six documents and whether these are explicit, included information but not detailed or not included. The ‘implementation process analysis’ focused on the details provided three main elements: the implementation plan, resources, and monitoring and evaluation (M&E), judged as critical to implementation (see more in Supplementary Annexe 2). By assessing coherence, incoherence is also assessed which can highlight the issues that decision-makers may face around understanding objectives and approaches that are cross-sectoral in nature.

To limit concerns such as validity, authenticity and bias, feedback from the primary author’s supervisors was received on interim outputs and analyses (Maxwell, 2016). The primary author acted as an ‘independent auditor’ to ensure a uniform approach to the collection of data and analysis contributing to validity and reliability.

## Results

The three analyses, a content analysis, cross-referencing maps and a policy coherence analysis, work together to tell a story of incoherence in climate adaptation and health policy in South Africa.

### Adaptation content across South Africa health and environment documents

Although South Africa has a National Climate Change and Health Adaptation Plan (NCHAP) and information on various health impacts related to climate variability exists, the characterization of adaptation varies by sector and governance level. Climate adaptations for health, referred to as health adaptations, consist of interventions or strategies that lessen adverse health outcomes and strengthen health systems against climate risks. The content analysis of 27 policy documents shows the ways in which climate change adaptation and specifically health adaptation are characterized in national and provincial health and climate-related documents by institutional author as summarized in Table 1 and further detailed in Supplementary Annexe 1.

**Table 1.** Holistic content analysis of climate change and health-related documents

Governance level	Sector	Number of documents	Inclusion and characterization of adaptation in document	Inclusion and characterization of health adaptation in document
National	Health	**7**	Adaptation is described in various ways, as environmental health and as necessary to address to avoid health impacts. Few documents do not include a description or discussion of climate change adaptation	Health adaptation is not clearly described in most documents. Some documents discuss adaptation scenarios for health, describe challenges in climate and health, the need for adaptation for the health sector or include specific health adaptations as it relates to a climate risk (such as heat)
National	Environment	**6**	The importance of adaptation for South Africa is described in most documents that include the challenges associated with adaptation and the progress and state of adaptation is described	Beyond health being named as a priority adaptation sector, health adaptation is not clearly discussed in the documents
National	Development/other	**3**	Adaptation is described in these documents in different ways; one document provides the Intergovernmental Panel on Climate Change (IPCC) definition. All documents include goals for adaptation as well as the limitations to adaptation	Health adaptation is not clearly discussed. There is only mention of all sectors, including healthcare, needing to address adaptation
Western Cape	Health	**3**	Adaptation is not discussed	Health adaptation is not discussed in most documents. There is only mention of adaptation plans under development in one document
Western Cape	Environment	**7**	Adaptation is described in a range of ways: as an opportunity for economic competitiveness, necessary for Western Cape climate response, to little description or discussion of adaptation	Health adaptation is not clearly discussed aside from little discussion of preventive public health measures for the health sector
Western Cape	Development	**1**	Adaptation is discussed as it relates to building capacity adaptive systems for a resilient economy and seen as a priority in every sector. The limitations to adaptation are also discussed	Health adaptation is not clearly discussed

### National level adaptation content

At the national level, adaptation is expressed differently across environment and health sector documents. Adaptation is recognized in environmental policy more than in health policy, and variation in the description of adaptation is especially visible in national health sector documents. For example, the NCHAP discusses adaptation as a short- to medium-turn solution to avoid projected health impacts of climate change, while the Health Strategic Plan does not make mention of climate change or include a discussion around adaptation. Yet, specific climate risks, like drought, are discussed in more detail in both the NCHAP and the Heat Health Action Guidelines (HHAG). Both documents also include specific adaptation strategies with illustrative activities.

In the environment sector, most documents include a discussion on the importance of adaptation, though few documents describe what adaptation is or provide a definition. For example, the National Climate Change Adaptation Strategy (NCAS) describes adaptation as an opportunity for South Africa to ‘transform both the health and the economy, to strengthen the social and spatial fabric, and to become more competitive in the global marketplace’, while the 2021 Climate Change Bill characterizes adaptation as important to address and relays requirements for adaptation planning and reporting (Department of Environment, Forestry and Fisheries, 2019). Interestingly, both national documents that are critical for the South African climate response, and specifically for adaptation, defer to one another as guiding adaptation for South Africa. The Climate Change Bill 2021 draft indicates that the NCAS will guide adaptation priorities for the country and result in the publication of Adaptation Reports by the Ministry of Environmental Affairs. Meanwhile, the current NCAS indicates that it is awaiting the Climate Change Act to define and provide a legislative framework for adaptation.

Foundational climate change documents, such as the NCCRP and the NCAS, are published by the environment sector, specifically the Department of Environment, Forestry and Fisheries, and name health as a priority adaptation sector that should engage and implement adaptation strategies. However, health adaptation is not clarified or defined in these documents. Some environment documents, including both governance level development plans, also discuss the limitations of adaptation.

Such limitations, better understood as barriers, relate to the challenges South Africa faces in assessing vulnerability in a standard way, financing adaptation activities and addressing cross-sector issues due to the involvement and administration of various government departments and spheres of government. The 2018 Third National Communication, a South Africa required United Nations Framework Convention on Climate Change report, reviewed a limitation to adaptation, that is revealing particularly for health. The document included that there is a lack of data on climate-health linkages, yet the same document expresses that there is enough information on linkages to make adaptation strategies for health. One can infer from this messaging that there are limited data available on South Africa specific risks to inform current adaptation strategies for health and that national action on adaptation, especially health adaptation, is challenging.

### Western Cape province adaptation content

At the Western Cape provincial level, there was acknowledgement by both environment and health sectors that adaptation is an important principle yet challenging to implement. Adaptation is not clearly characterized in provincial health sector documents, signifying an absence of adaptation considerations in provincial health sector strategies. These documents used similar language across different years to describe the importance of addressing climate change for the health sector but do not include a description of adaptation. The Western Cape Health Strategic Plan 2020–2025 and 2014 visionary Western Cape health sector document, HealthCare 2030 (HC2030), mention that adaptation plans and strategies are under development. Yet, in the 2022 Western Cape Climate Change Response Strategy (WCCRS), the health sector did not include a health sector-specific adaptation plan, suggesting a delay in adaptation plan development despite progress being reported.

In environment sector documents, the purpose of adaptation is inconsistently described. In the Biennial Monitoring and Evaluation Report, adaptation is seen as an opportunity for economic competitiveness and in the WCCRS adaptation is seen as necessary for Western Cape climate response. The one development document authored by the Office of the Premier, the Western Cape Provincial Strategic Plan, includes a discussion of adaptation that relates to building overall capacity adaptive systems for a resilient economy. Although illustrative actions or preventative measures are included in the WCCRS, health adaptation is not clearly discussed in any of the 11 Western Cape provincial documents.

### Coherence of documents to support implementation

**Figure 1.**
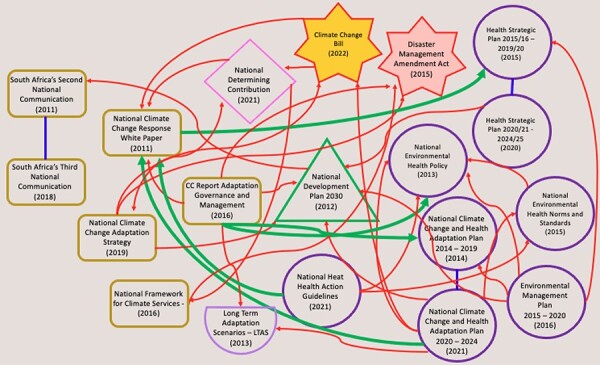
Document referencing by institutional author at the national level

**Figure 2.**
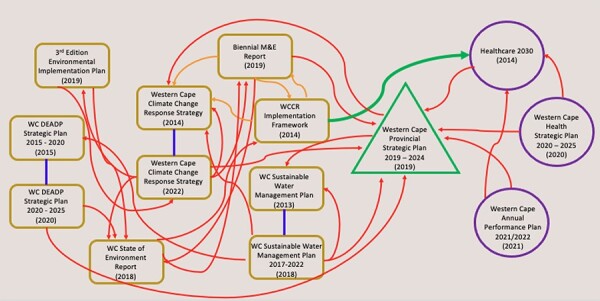
Document referencing by institutional author at Western Cape provincial level

The findings of the coherence analyses are illustrated with two figures: Figure 1 that shows linkages, or horizontal coherence, between national documents and Figure 2 between provincial level documents. Documents were organized by sector and cross-sector references within a governance level and were indicated by a bolded arrow.

Key: Boxes—environment sector; circles—health sector; triangle—Department of the Presidency. Arrows indicate referencing of a document. Bolded arrows indicate a cross-sector reference.

Key: Boxes—environment sector; circles—health sector; triangle—Department of the Premier. Arrows indicate referencing of a document. Bolded arrows indicate a cross-sector reference.

### National documents of significance

The seminal NCCRP is referenced several times by health sector documents such as the National Climate and Health Adaptation Plan (NCCHAP) and the HHAG, the first heat plan for South Africa, as seen in Figure 1. The NCHAP references the NCCRP as a document it is taking into consideration under the new iteration of the plan, and the HHAG names the document as local legislation with which the guidelines align. The cross-reference from the NCCRP to the Health Strategic Plan was a general reference that the Department of Health’s Strategic plan (no specified year) will identify adaptation needs and interventions, integrate adaptation strategies and coordinate across sectors on adaptation response.

South Africa’s NCAS provides guidance on adaptation actions for South Africa by priority sector, including the health sector but it is not referenced in health policy documents after 2019.

### Development plan cross-referencing

Most documents reviewed at both national and provincial levels link to the relevant governance level’s development plan, that is the Provincial Strategic Plan and the National Development Plan (NDP), that act as long-term guiding documents for cross-sectoral priorities which is especially critical for meeting objectives in the two adaptation documents NCAS and NCHAP (Figures 1 and 2). The NDP has a dedicated chapter on adaptation with the goals of implementing adaptation strategies and investing in adaptation technologies. The Western Cape Strategic Plan names adaptation as a priority in the health sector and as critical in order to reduce risk exposure and ensure human well-being and social development. Both documents acknowledge adaptation but, like other national and provincial level documents, are not coherent when it comes to health adaptation. Although development plans guide the respective governance level sectoral planning, references to the development plan are to simply align the document purpose and objectives with that of the governance level development plan. This intra-governance level alignment of priorities and objectives may not emphasize larger country priorities, such as climate response priorities or a vision to act on climate adaptation.

### Cross-referencing within and across sectors at national and provincial level

Both maps show that cross-referencing across health and environment sectors within each governance level is quite limited. Instead, there are many intra-sectoral references that highlight siloed working within sectors. Such siloed working also happens between governance levels. For example, the NCHAP, authored by the national Department of Health, is not referred to in health sector documents at the provincial level. This siloism might be present because according to climate change documents the environment sector is called on to develop and lead the climate change agenda in South Africa.

At the Western Cape level, only one cross-sectoral reference is made from the environment to the health sector. This is in the 2014 WCCRS implementation framework that lists HC2030 (the health strategic vision document) as a contextual reference, commenting on the importance of health communities to monitor health and climate trends in the Western Cape. At the national level, the health sector has more climate-related documents such as the NCHAP and HHAG that cross-reference environment sector documents more than other health sector documents.

Environment sector-authored documents at both the national and Western Cape level include climate-related policies and plans that include intra-sectoral referencing and little referencing of health sector documents. The sector-authored documents also include the Climate Change Bill and the National Determining Contribution, as the role of the environment sector in climate change is much more defined and has led to the sector leading on key climate legislation, budgets and committees.

The NCCRP is the most cross-referenced plan that was the first South African document to relay health adaptation information. At the Western Cape level, HC2030 was the only cross-referenced provincial health sector document and, although not clearly specified, the document included health adaptation information. Both documents were included in the policy coherence analysis.

### Policy coherence of key documents

While policy integration looks at inputs and goals and the procedures and institutional arrangements that influence policy-making, policy coherence looks at policy outputs, objectives, instruments and implementation (Nilsson *et al*., 2012). Six documents, four at national and two at Western Cape level, contain health adaptation content and were the most frequently cross-referenced documents. The subsequent policy coherence analyses consider the extent to which these documents include features relevant to implementation, specifically, measures of health adaptation and implementation process.

### Measures for health adaptation

An optimal adaptation measure in a policy or strategy should aim to achieve its intended outcomes, with an M&E framework in place to ensure continuous adaptive management and improvement, including associated indicators (Bo *et al*., 2005; Berrang-Ford *et al*., 2011). Health adaptation measures need to mitigate current risks and anticipate the effects of climate change to prevent and limit adverse health outcomes (Mehta and Luber, 2009; Luyten *et al*., 2023). The list of health adaptation measures in key documents can indicate if a policy can be useful for adaptation. All documents include a form of a health adaptation measure, while the NCAS, NCHAP and HHAG include explicit health adaptation measures with measurable indicators. This coherence analysis differs from the content analysis where health adaptation content including evidence, strategies or expressions of urgency around climate change adaptation to improve health were assessed. For the purposes of the coherence analysis, health adaptation measures were characterized in documents as actions, goals, outcomes or elements and included the health sector or a health institution as a lead of the measure.

Table 2 presents the health adaptation measures in each of the six key documents and assigns a rating to each document to show which documents contain explicit and detailed health adaptation measures often with indicators or expected results or health adaptation measures that are informative but lack detail by only relaying goals or illustrative activities. The following ratings are used to assess the inclusion of information in each of the documents: E for explicit and detailed inclusion, I for included information but not detailed and X for not included.

**Table 2.** Measures for health adaptation

Document name and year	Rating	Notes	Health adaptation measures
**NCCRP (2011)**	I	No indicators provided. Listed health-specific actions	**Objectives**: ‘**5.4.1** Reduce the incidence of respiratory diseases and improve air quality through reducing ambient particulate matter, ozone and sulphur dioxide concentrations by legislative and other measures to ensure full compliance with National Ambient Air Quality Standards by 2020. In this regard, the use of legislative and other measures that also have the co-benefit of reducing Greenhouse gas (GHG) emissions will be prioritized. Progress in this regard will be published on the South African Air Quality Information System. **5.4.2** Recognizing that the nutritional status of individuals is key to building resilience to environmental health threats, ensure that food security and sound nutritional policies form part of an integrated approach to health adaptation strategies. **5.4.3** Develop and roll-out public awareness campaigns on the health risks of high temperatures and appropriate responses including improved ventilation and promotion of behaviours that minimize exposure to high temperatures, namely “avoidance behaviour”. **5.4.4** Design and implement “Heat-Health” action plans including plans in respect of emergency medical services, improved climate-sensitive disease surveillance and control, safe water and improved sanitation. **5.4.5** Strengthen information and knowledge of linkages between disease and climate change through research. **5.4.6** Develop a health data-capturing system that records data both at spatial and temporal scales and that ensures that information collected can be imported into multiple-risk systems such as the South African Risk and Vulnerability Atlas. **5.4.7** Improve the bio-safety of the current malaria control strategy. Although the current strategy, which includes the use of the persistent organic pollutant, Dichlorodiphenyltrichloroethane (DDT), has proven effective in reducing the incidence of malaria, there are significant concerns about its long-term impacts on environmental and human health. **5.4.8** Strengthen the awareness programme on malaria and cholera outbreaks.’
**NCAS** (**2019**)	**E**	Included indicators. Outcomes where Department of Health is lead	**‘1.1.8** Launch an enhanced climate change public flagship programme to build a healthier, more resilient society **1.1.9** Equip and capacitate healthcare facilities to manage climate change-related health effects and climate-sensitive diseases’
**NCHAP (2020)**	**E**	In draft. Lists activities to support adaptation and lists expected results and outputs by activity	**‘Component 1: Establishment of a structure for the co-ordination of national action plan**. Expected Result: Structure in place, tools and technical support available **Component 2: National vulnerability and health system assessments** Expected Result: National vulnerability assessment (VA) conducted **Component 3: Capacity building** Expected Result: National core capacities for the sound management of public health risk related to climate change made available **Component 4: Integrated environment and health surveillance** Expected Result: Timely, evidence-based decisions are taken for the sound management of public health risks related to climate change **Component 5: Response** Expected Result: Reduction in public health impact of climate change **Component 6: Research** Expected Result: Local knowledge on climate change health risk factors, their management and indigenous adaptation strategies documented and disseminated **Component 7: Monitoring and evaluation** Expected Results: Programme implemented effectively and in a timely manner; Process, result and impact indicators of the programme assessed, documented and disseminated; Annual progress reports **Component 8: Management and coordination** Expected Result: Programme expected results achieved’
**HHAG (2022)**	**E**	Included expected results	**‘Short term**: ∼Assessment of heatwave definition and then finalise a set of messages about heat and health. ∼Assessment of health system preparedness for heat-health impacts ∼Activate heat health warnings with heat wave warnings ∼Heat health material into school curricula ∼Develop curriculum material based on South African evidence ∼Develop and implement heat-health (climate change) indicators **Medium term**: ∼Set a temperature threshold above which sports events, school sports etc. will not be permitted to occur. Such events would need to be postponed and re-scheduled during cooler weather. ∼Assess state of adequate and ease of access to drinking water, hygiene and sanitation in schools especially during extreme heat and heatwave (overcrowding in classrooms exacerbates heat-health impacts).∼Review school classroom thermal comfort. ∼Review healthcare facilities thermal comfort. ∼Implement climate change indicators Surveillance activity met.
			**Long term**: ∼Review school uniforms for heat health implications. ∼Review regulations around personal protective equipment for outdoor occupational workers. ∼Urban planning to provide heat interventions.’
**HC2030** **Road to Wellness** (**2014)**	I	No indicators provided. Listed adaptations of what health service intends	**‘Climate-related goals**: Improved surveillance and disease outbreak management capacity; better disaster management and rescue responsiveness in collaboration with other departments; and strengthened emergency services within health’
**WCCRS (2022)**	I	No indicators provided. Goals that include health but not for health sector	**‘Health-related goals**: ∼Adapt our health systems to the realities of a harsher climate and increased vulnerabilities, focusing on bolstering the capacities and climate awareness of community health worker networks and making sure that early warning systems improve health sector responses to extreme events ∼Lobby for social protection policies and climate adaptation policies that extend to food security and mental health support’

### Health adaptation measures in national and Western Cape documents

Explicit and detailed health adaptation measures were included in the NCAS, NCHAP and the HHAG, all of which are national documents. The NCAS environment sector document lists several adaptation measures—with two that are explicitly health adaptation measures led by the health sector.

In the HHAG, explicit measures are offered as actions, including timeframes, expected results and participating institutions such as the short-term measure seen in Table 2 ‘Develop and implement heat health (climate change) indicators’. The expected result of this measure is to increase surveillance of heat/health impacts. As a health sector-led policy, the health sector is ultimately accountable for the expected results of the guidelines; however, as further discussed in the ‘implementation process analysis’, these expected results are not accompanied with a framework for reporting to track progress. Finally, both Western Cape documents do not include explicit health adaptation measures or indicators. Instead, they include broader goals such as adapting health systems to climate change.

### Coherence of national health adaptation measures

Of the three national documents that included explicit health adaptation measures, there are instances of coherence among the measures. One such example is around climate and health programmes and health system activities. In Table 2, a relevant NCAS measure 1.1.8 to start up a programme focused on climate change is coherent with Component 2 in the NCHAP where established implementation nodes in provinces and municipalities are tasked with carrying out climate programmes for health. Similarly, NCAS measure 1.1.9, assisting health care facilities to address climate-related diseases and health effects, aligns with NCHAP Component 3 around healthcare sector capacity and Component 4 on integrating surveillance for climate-sensitive diseases. These are instances of coherence across an environment sector document and health sector document.

Significantly, there is coherence around health adaptations as they relate to heat as heat planning is the most progressed. Both the HHAG and NCHAP align with measures in the NCCRP such as NCCRP 5.4.4 ‘Design and implement “Heat Health” action plans…’. NCCRP 5.4.3 is particularly aligned with the HHAG as it relates to raising public awareness on the health risks of heat and high temperatures and relay actions and responses the public should take to protect their health.

### Implementation process

The health adaptation implementation process is assessed by looking at horizontal policy coherence amongst documents at each governance level on key implementation elements (Nilsson *et al*., 2012). The assessment reveals whether roles and responsibilities are stated in key documents to guide health adaptation, if resourcing was considered, as well as whether implementation plans include frameworks to track progress and improve actions. Implementation process elements are further described in Supplementary Annexe 2. Table 3 shows whether each document included these key implementation elements and the degree to which they are detailed, to determine if the documents can inform and support implementation of health adaptation strategies. The following ratings are again used to assess the inclusion of information in each of the documents: E for explicit and detailed inclusion, I for included information but not detailed and X for not included.

**Table 3.** Implementation process elements in documents for health adaptation

	**Implementation plan elements**			**Allocated resources for implementation**		**M&E**		
**Document name**	**Institutional set-up**	**Allocation of responsibilities**	**Timeline**	**Financial**	**Human**	**Framework and reporting**	**Lessons learned**	**Notes**
**National Documents**								
**NCCRP** (**2011)**	I	I	I	I	X	I	X	States that within 2 years of policy South Africa will publish Climate Change Response Monitoring and Evaluation System
**NCAS** (**2019)**	**E**	**E**	I	I	**E**	**E**	I	Lessons learned are said to be part of an M&E system that will be put in place as one of the objectives
**NCHAP** (**2020)**	**E**	I	I	I	X	**E**	I	Vague timeline (most in past) and given arbitrary budget numbers included
**HHAG** (**2022)**	**E**	I	I	X	X	I	X	Actions for implementation in an Annexe separated from discussion of M&E and Lessons
**Provincial documents**								
**HC2030** (**2014)**	X	X	X	X	X	X	X	Does not include implementation elements
**WCCRS** (**2022)**	I	X	X	I	X	I	I	Includes M&E section that discusses intentions and principle of learning to support local climate response

### Implementation process supported in national documents

Table 3 shows that there is, overall, limited information in the documents about key implementation elements related to health adaptation, such as roles and responsibilities, resourcing, and M&E. This is especially the case for health sector documents at both governance levels. The three documents that include explicit information on implementation process elements, the NCAS, NCHAP and HHAG, showed varied inclusion of implementation process elements. The NCAS provided explicit information on institutional actors and their roles; however, it did not provide specific timeframes for actions nor did it provide an associated budget. Similarly, the NCHAP includes a vague timeline for actions and an illustrative budget allocated by year. The process elements ‘Timeline’ and ‘Financial Resources’ were weak across all six documents. This can indicate potential limitations of policy documents to effectively include critical implementation elements that require additional context and planning across a department’s competing priorities and strategies.

Although the NCHAP has explicit measures for health adaptation, like the NCAS, it does not include information on key implementation process elements such as institutional information, clarity in allocation of responsibility and a timeline—all of which are critical for accountability and action on the implementation of health adaptation actions. Institutional arrangements for implementation stated in the NCAS and the NCHAP differ from the HHAG where a lead is named along with partners, setting up a form of accountability. Although institutions are named in the HHAG, all the institutions are grouped together. This places uncertainty on who is the lead and responsible institution and actors are to carry out an adaptation measure. This further differs from the NCCRP and WCCRS that name the entirety of the health sector under a comprehensive health-focused section of the document, leading to a lack of accountability among health sector institutions and actors.

The two Western Cape provincial policies examined do not have explicit implementation process elements and are severely lacking the detail that can support implementation. The lack of implementation elements in Western Cape documents might suggest that action on adaptation for health is not occurring or is occurring outside formal policy directives.

## Discussion

There are few case studies of policy coherence in climate and health, and this paper presents one of the first detailed analyses of cross-sector and multi-level coherence between environment and health sector climate change adaptation policies, focusing on South Africa. Our findings show that there is limited coherence across either sector or governance level, and therefore, existing policy documents do not appear to provide a clear foundation for effective implementation of health adaptation responses to climate. Of the documents reviewed, national documents include a few aligned health adaptation measures, some of which relate to health adaptation to heat.

Existing research showing connections between climate exposures and health impacts notes that health is not sufficiently addressed or mainstreamed into national, subnational and local climate adaptation plans (Orru *et al*., 2018; Nhamo and Muchuru, 2019; Woodhall *et al*., 2019; Aracena *et al*., 2020). Mainstreaming in this context is the inclusion of climate and health strategies, related information and indicating climate and health as a priority. While an important first step, our findings show that the intent to ‘mainstream’ or align cross-sectoral priorities such as climate change does not necessarily result in coherent policies that support implementation. Except for the 2011 NCCRP, ‘mainstreaming’ in the reviewed documents often seems to be accomplished as little more than the inclusion of the term ‘climate change’ and its risks to health or society. This narrow definition is supported by South African national reporting where ‘mainstreaming’ of climate change response, including mitigation and adaptation, was reported as achieved in 2018/2019. It does not include any reporting on whether a document’s objectives take climate change into account or whether they outline essential implementation elements.

The policy incoherence documented in this study could be due to sectoral siloism—common in both policy formulation and implementation—or the autonomy of subnational actors. Although some scholars have acknowledged the importance of vertical working in formal institutional structures, silos have notably contributed to a lack of shared information, delayed and ineffective decision-making as well as the inability to resolve ‘wicked problems’ that affect a multitude of sectors and departments (Jurkiewicz, 2009; Bezes *et al*., 2013; Peters, 2015). A lack of effective coordination between government departments has contributed to unsuccessful horizontal working (Scott and Gong, 2021). Climate risks should be managed and adapted cross-sectorally, yet this is difficult to realize in practice. Working in silos ultimately acts as a barrier in achieving cross-sector objectives and goals, as noted in previous literature (Godsmark *et al*., 2019; Aracena *et al*., 2020). Coordinating mechanisms, such as those proposed in the 2020 National Public Health Institute of South Africa Act which aims to improve public health service delivery and coordination of national disease surveillance systems, could improve coordination across sectors on climate adaptation for health. It could also be argued that a One Health framing can offer a coherent approach to work across health, environment as well as animal health although the documents reviewed did not consider One Health in relation to climate adaptation. Ultimately, it is the actors responsible for implementation who can shape the way in which policies are delivered in practice (Hupe and Hill, 2016). Their decisions are nuanced and dependent on systems and institutions, influencing the implementation of the objectives set out in formal policy documents that can further explain the incoherence of policies reviewed, particularly at the Western Cape provincial level.

It seems that there is limited cross governance level engagement in policy development of climate change adaptation, especially in the health sector, that can impact climate action. South Africa has a devolved public governance system in which policy is developed across governance levels to allow for region-specific priorities and account for local context. Therefore, although national documents set policy priorities and a vision for the South African climate response, lower governance levels, such as the provincial level, must include information and detail in their policy documents to support the implementation of national priorities (The Republic of South Africa, 1996). Aracena et al. (2020), in their Peruvian case study of the health system adaptation to climate change, disclose that the NCCHAP did not provide a clear designation of responsibility which led to an absence of climate and health adaptation strategies at the provincial level (Aracena *et al*., 2020). Beyond environment and health sector policies, development plans should also integrate meaningful climate change adaptation planning to support the alignment of institutional objectives and priorities. The development plan is a critical document for each governance level’s alignment of priorities and has influence on future budget line items—which is critical to solve the under-resourcing challenge of work on climate adaptation (Huang *et al*., 2011; Orru *et al*., 2018).

At the provincial level, the Western Cape health sector has taken steps to strengthen health system resilience to all risks. For example, improved surveillance, the use of data for decision-making and the development of distributed leadership supported the Western Cape’s COVID-19 response (Quintana *et al*., 2023). Although this health system strengthening approach is also critical to a health adaptation response and health systems must adapt to climate risks and work towards resilience, a resilience to all risks approach can undermine the extent to which climate risks are considered in health strategies. The incoherence among Western Cape health sector documents might also suggest that decision-makers may not have sufficient information or understanding of health adaptation at the subnational level. The failure to identify responsible entities or departments in national plans and a lack of adaptation strategies in subnational policies results in poor accountability of adaptation activities, lack of resourcing and guidance for decision-makers, as well as inevitable gaps in implementation.

The Western Cape province encourages a whole of society—whole of government approach to addressing challenges to sustainable development (Western Cape Government, 2019; Western Cape Government Health, 2021). Like the ‘mainstreaming’ discussion earlier, this might provide further insight as to why institutional policies are all encompassing at the provincial level and appear to not address health adaptation. If all challenges must be acknowledged in all policies, there is an encouragement of mainstreaming all country priorities, like climate change, and there is then little room to provide depth on needs and for a sector to take full ownership of climate adaptations for health. The utilization of existing institutional arrangements, such as government clusters and fora like the municipality support programme that address cross-cutting issues, can promote an integrated approach to policy-making and planning, and ultimately decision-making, for climate change and health.

Our findings show that although health adaptation measures are somewhat coherent in national level policies, implementation elements such as resourcing and M&E are barely considered in these documents. Given the devolved South African governance structure, the absence of implementation elements in national documents is perhaps not surprising. Despite this, the NCAS is the document that includes the most explicit implementation process elements for health adaptation including allocation of responsibilities and an M&E framework. Interestingly, the NCAS is not a health sector document. This puts implementation of health adaptation into question. Do health decision-makers receive direction for health adaptation from non-health sector documents? Do decision-makers outside the health sector work on health adaptations? Our analysis of subnational documents—the level at which implementation activities occur for health—suggests that the answer to these questions may be ‘no’. The inconsistencies of health adaptation across Western Cape provincial documents do not offer sufficient or coherent guidance to support decision-makers in implementation. This can further suggest that health adaptation is not guided by policy and can be occurring outside of policy objectives.

## Limitations

Policy documents offer a limited perspective on actions, events and behaviours that occur in real time, and therefore, the findings from this policy document analysis are not all encompassing. In addition, there may be updated versions of the documents since analysis was conducted. Given that South African provinces have constitutional responsibility for health service delivery, including management of the provincial health budget, this subnational level is critical to understand in examining decisions on health adaptation. As this policy document analysis did not include local government policy, the third tier of South African government, this is a limitation of this paper.

## Conclusion

Overall, there is incoherence in South African climate adaptation for health policies at both the national and Western Cape provincial level. The meaning of adaptation, especially health adaptation, is not clear across documents, except for health adaptation to heat. While South African health sector documents largely commit to mainstreaming climate change adaptation, they do not include specific elements that are critical to support implementation. This might be due to the cross-cutting nature of climate change and the distinct responsibility placed on the health and environment sectors in managing climate risks in their own portfolios.

As a cross-cutting issue, climate change should move beyond mere acknowledgement in sectoral plans towards a tailored response plan of actions that are geographic and sector specific. It would be helpful for the South Africa health sector to develop further climate risk-specific policies such as the National HHAG, especially at the subnational levels where implementation of health adaptation activities is coordinated and managed. Such climate risk-specific documents for the health sector would support the implementation of health adaptation and contribute to building the health system’s resilience.

## Supplementary Material

czae011_Supp

## Reflexivity Statement

The first author is a self-funded international student who is a white-facing Latina. While in South Africa, the first author was conscious of her social positionality given the recent apartheid history that is still felt in Cape Town. Furthermore, the first author disclosed her American nationality that provided some removal from the British history London School of Hygiene and Tropical Medicine’s affiliation comes with. The first author acknowledges that she is not South African and therefore is at a disadvantage of understanding complexities and nuances in South Africa. As a non-South African, the first author brings an unbiased perspective to this research. The first author’s affiliation with LSHTM and University of Cape Town provided her with an advantage where there is recognition of accolade and trust. This paper is part of the first author’s thesis project, where her two supervisors and in-country advisor are co-authors of this original journal article.
