# Supplementary material for: A story of (in)coherence: climate adaptation for health in South African policies
Source: Health Policy Plan. 2024 Feb 28;39(4):400–11. doi: 10.1093/heapol/czae011 (PMC11005833; doi:10.1093/heapol/czae011)
Supplement: czae011_Supp [file czae011_supp.zip › supp/Supplementary Info.docx]

**Annex 1**

**Table 1: National Documents Climate Adaptation Content**

| **Document Name and Year** | **Institutional Author(s)** | **Adaptation Content** | **Health Adaptation Content** | **Climate Risks Identified** | **Health Impacts Identified** |
| --- | --- | --- | --- | --- | --- |
| National Climate Change Response Policy White Paper (2011) | Department of Environment, Forestry and Fisheries (DEFF) | There is a strong emphasis on the importance of adaptation for South African Climate response that encourages more GDP spending on adaptation and acknowledges adaptation responses are stronger at a local context than mitigation responses. | Health is named as a priority adaptation sector. Adaptation strategies are said to be integrated into the Department of Health Strategic Plan with illustrative health adaptations. | Limited climate risks information throughout document. No evidence used. | Some information on health-related impacts discussed - vector-borne diseases and incidence of water-borne diseases |
|  |  |  |  |  |  |
|  |  |  |  |  |  |
|  |  |  |  |  |  |
|  |  |  |  |  |  |
|  |  |  |  |  |  |
|  |  |  |  |  |  |
|  |  |  |  |  |  |
|  |  |  |  |  |  |
| National Development Plan 2030 (NDP) (2012) | Department of the Presidency | Adaptation is said to improve economic and societal resilience. A section in the plan is dedicated to adaptation where one of the goals is to have adaptation strategies actively piloted, implemented, and invested in by 2020. | Health adaptation is not clearly discussed. Instead, there is mention that all sectors will be more resilient to climate change and improving healthcare is raised. | Mention of climate risks such as increased extreme rainfall events and drought. | Health impacts not explicitly discussed aside from infectious disease risks as a result of climate change. |
| National Environmental Health Policy (2013) | Department of Health | Adaptation is described as environmental health is key to the climate response through the consideration of environmental health in adaptation planning. | Health adaptation is not clearly discussed. The policy mentions the health sector is participating in climate change as it relates to energy efficiency within health facilities, public awareness, and education. | No discussion of climate change risks. | Environmental health information discussed such as environmental-related disease due to contaminated water, poor hygiene, inadequate sanitation. Acknowledgement of the link climate change plays in exacerbating impacts. |
|  |  |  |  |  |  |
| Long Term Adaptation Scenarios: Health (2013) | Department of Health | Adaptation focused document that aims to respond to the National Climate Change Response Plan from 2011 for the health sector | Health adaptation is discussed as national and sub-national adaptation scenarios for the South African health sector under possible future climate conditions and development pathways. | Information on rainfall and high temperatures is presented with evidence | Nine health focuses areas are discussed as it relates to climate change |
| National Environmental Health Norms and Standards (2015) | Department of Health | Adaptation is not discussed. Instead, it lays out the roles and responsibilities of Environmental Health Practitioners (EHPs) where it is not clear if climate adaptation is the responsibility of EHPs | Health adaptation is not clearly discussed although responsibilities of EHPs listed can serve as health adaptation actions | Climate risk of flooding taken into consideration | Information on environmental health but limited in linking to climate change. Information provided does not use evidence. |
|  |  |  |  |  |  |
|  |  |  |  |  |  |
| Disaster Management Amendment Act (2015) | Department of the Presidency | This amendment to the Disaster Management Act adds climate change and adaptation. Adaptation is defined using the IPCC definition. The Act strengthens the requirement of organs of states to address adaptation that includes the national, provincial, and municipal level to conduct disaster management plans and work out how to invest in disaster risk reduction and climate adaptation. | Health adaptation is not discussed. | No discussion of climate change risks. | No health impacts as it relates to climate change discussed |
| National Framework for Climate Services (2016) | Department of Environment, Forestry and Fisheries (DEFF) | Adaptation appears as a central concept for this document as the activities in the document aim to improve the country’s weather and climate forecasting and prediction capabilities, services, and applications to address adaptation to climate variability and change. | Health adaptation not clearly discussed. There is mention that the framework could produce sector specific products, for health where the use of climate information for health can anticipate incidence of diseases, and estimate possible health consequences of climate risks to help public health services with warnings and planning | Climate risks, such as drought and flood are discussed | Information on use of climate services for can be used for (malaria) outlooks and to provide early warning for public health. |
| First Annual Climate Change Report: Adaptation, Governance, and Management (2016) | Department of Environment, Forestry and Fisheries (DEFF) | An overview of progress made on climate adaptation governance and management until 2016 is given. A 2015 situational analysis and needs assessment found gaps in climate adaptation coordination, among other items | Health adaptation is described through existence of an adaptation strategy working through implementation with ministerial approval. The 2014 to 2019 National Climate and Health Adaptation Strategy is compared alongside other sector adaptation strategies where key adaptation options from the plan are listed. Institutional coordination on climate change adaptation for the health sector are listed. | No discussion of climate change risks. | No specific health risks discussed |
|  |  |  |  |  |  |
|  |  |  |  |  |  |
| National Environmental Management Plan 2015 – 2020 (2016) | Department of Health | Adaptation is not discussed in depth. This document acknowledges the role the health sector plays in adapting to climate change. | Health adaptation is not clearly discussed. Climate and Health is listed as an environmental management function of the DOH and activities, targets and indicators are included to address cooperative governance on the implementation of the Climate and Health Adaptation Plan commitments | Mention of climate risks but no evidence provided | Mention of health impacts of climate change listed with no evidence provided |
|  |  |  |  |  |  |
|  |  |  |  |  |  |
| South Africa’s Third National Communication (2018) | Department of Environment, Forestry and Fisheries (DEFF) | The state of adaptation in South Africa is discussed where a need for dedicated financial resources for climate adaptation planning and implementation at a provincial and local level with guidance is expressed. There is mention of the development of an M&E-system led by DEA for mitigation and adaptation aimed to provide an evidence-base of climate change in South Africa and associated responses | Health adaptation is not clearly discussed. The health sector has the least projects in adaptation compared to other priority adaptation sectors. | Only wildfires as a climate risk are discussed as well as information on projections for rainfall and temperature. | A summary of the vulnerabilities to climate change for the health sector is included which stated the health sector is in a vulnerable state with quadruple burden of disease, poor housing, distribution of disease, and health impacts due to water supply and agriculture. A limited understanding on climate and health linkages is expressed |
| National Climate Change Adaptation Strategy (NCAS) (2019) | Department of Environment, Forestry and Fisheries (DEFF) | As the adaptation document for South Africa, adaptation is described as an opportunity for South Africa ‘to transform both the health and the economy, to strengthen the social and spatial fabric, and to become more competitive in the global marketplace’. The Strategy outlines key strategic goals and outcomes needed to respond to climate change with adaptation and outlines the vision and strategic goals. | Health is listed as a priority adaptation sector. The Strategy does not go into depth as to how adaptation will occur in the health sector, but does include health led adaptation activities | Limited information provided on climate risks only mention of fire, floods, heat, and drought. | Information on health as it relates to climate included throughout document, presenting evidence. There is an inclusion of the need for a Health Sensitivity Analysis and Health Exposure Analysis, and mention of adaptation work by the health department such as an early warning system for Malaria |
| Health Strategic Plan 2020/21 - 2024/25 (2020) | Department of Health | This document makes no mention of climate change or climate adaptation. | No mention of health adaptation | No discussion of climate change risks. | Health focused, but not as it relates to climate change. |
| *Draft* National Climate Change and Health Adaptation Plan 2020 – 2024 (NCHAP) (2021) | Department of Health | Adaptation is seen as a short- to medium-tern solution to avoiding projected health impacts of climate change while mitigation is seen as a necessary long-term action to avoid further degradation of environmental determinants of health. | This is the third iteration of a health sector adaptation plan for South Africa that characterizes challenges in climate and health adaptation and proposes steps that may be taken by the health sector. | Information presented on heat stress, dry spells, sea level rise, floods, wildfires, disasters. Examples for drought and heat risks were Western Cape and Cape Town specific. Information on other climate risks from Japan and US | Information impacts on health were discussed. Suggested adaptation activities are written in illustrative form from capacity building, monitoring and surveillance, to health impact assessments and indicator development. |
|  |  |  |  |  |  |
|  |  |  |  |  |  |
| Climate Change Bill (2021) | Parliament of South Africa - DEFF | This Bill, originally drafted in 2018, does not describe adaptation instead it relays the importance of adaptation responses and includes requirements for adaptation planning. The National Adaptation Strategy is said to include adaptation scenarios that will result in the publication of Adaptation Reports for the cabinet and to respond to national and international processes | Health adaptation is not clearly described. The health sector is listed as responsible for developing a sector adaptation strategy and plan. | No discussion of climate change risks. | No specific health risks discussed. The right to an environment that is not harmful to health and well-being is acknowledged and health is seen as a functional area for climate response |
| National Determining Contribution (NDC) (2021) | Presidential Climate Commission (PCC) | The NDC represents the broader vision for adaptation planning. The overall aspirations for adaptation are outlines along with timelines and levels of investment needed to achieve the goals. The document highlights that subnational level institutions are key implementers of adaptation-related programs and projects. There is acknowledgement that they lack the capacity to lead local adaptation. | Health adaptation is not clearly discussed. The document relays that health is considered one of the most important adaptation sectors as well as focal areas to prioritize within health. | No discussion of climate change risks. | Minimal health information provided, such as climate induced diseases, climate impacts on health systems and climate change related epidemics. |
| National Heat Health Action Guidelines (HHAG) (2022) | Department of Health | This is an adaptation document that focuses on extreme heat risks and looks at interventions at the individual and community level that help people adapt to high temperatures. | Health adaptations are described in depth. Heat Health Warning Systems are seen as an important adaptation strategy to manage health risks associated with extreme heat and heatwaves. There is also mention of EHPs role in climate response and adaptation implementation | Only discussed heat as a climate risk in depth, with information, evidence, stakeholders, and heat targets. Wildfires briefly discussed. | Extensive information on health impacts as well as health system impacts. |
|  |  |  |  |  |  |
|  |  |  |  |  |  |

**Table 2: Western Cape Documents Climate Adaptation Content**

| **Document Name and Year** | **Institutional Author(s)** | **Adaptation Content** | **Health Adaptation Content** | **Climate Risks Identified** | **Health Impacts Identified** |
| --- | --- | --- | --- | --- | --- |
| Healthcare 2030 (HC2030) (2014) | Western Cape Department of Health | Adaptation is not discussed. A short climate section that is not integrated into the rest of the document | Health adaptation is not clearly discussed. There is mention that adaptation plans are under development at both national and Western Cape Government health level | No climate risks discussed only acknowledgement of extreme weather events being a risk. No evidence provided. | No health information provided as it relates to climate change |
| WCCRS Implementation Framework (2014) | Department of Environmental Affairs and Development Planning (DEADP) | Adaptation is important to Western Cape climate response, needing enabled locally effective adaptation action. The framework accompanies the previous iteration of the WCCRS and seeks to combine climate adaptation and mitigation in implementation where possible. | Health adaptation is not clearly discussed. There is mention of monitoring health parameters in relation to climate that would allow for the development and implementation of timely interventions. Maladaptation is raised as a concern when a sectoral view is taken. | No discussion of climate change risks. | Limited health information is discussed. The Healthy Communities strategy names a priority of monitoring climate and health trends, specific to Western Cape. No evidence is provided. |
|  |  |  |  |  |  |
|  |  |  |  |  |  |
| WC Sustainable Water Management Plan 2017 - 2022 (2018) | Department of Environmental Affairs and Development Planning (DEADP) | This document sees adaptation as an important principle as it should be applied across society to protect water. The adoption of the plan by other departments is seen as a climate adaptation measure. | Health adaptation is not discussed. | Mention of climate change impacts on water stress through increasing temperatures, evaporation, and extreme events such as droughts, fires, and floods. | No information on health impacts of climate change, just from lack of water access. Health discussed in context of river catchments and the environment |
| Western Cape State of Environment Report (2018) | Department of Environmental Affairs and Development Planning (DEADP) | Adaptation is not clearly discussed. Mentions of adaptation are around challenges behind implementation. Local climate change adaptation plans are mentioned as a key action for building resilient communities. | Health adaptation is not discussed | Information on projected climate trends and climate risks discussed. | Limited information on health provided, only acknowledgement that there are impacts on health. No evidence is presented. |
| Western Cape Provincial Strategic Plan 2019 - 2024 (2019) | Department of the Premier | Adaptation is described in the context of building capacity and systems ability to adapt to shocks or stresses for a resilient economy. | Health adaptation is not clearly discussed. Adaptation is seen as priority in every sector and critical to reduce risk exposure and ensure human well-being and social development | Information on floods, heat, fires, and drought with no evidence provided | Climate and health links are identified as it relates to a healthy community. No evidence provided. |
| Biennial Monitoring and Evaluation Report (2019) | Department of Environmental Affairs and Development Planning (DEADP) | Adaptation is seen as an opportunity to increase economic competitiveness of the Western Cape and necessary to economic development. Adaptation work and activities in South Africa are to occur at municipal levels | Health adaptations are not clearly described. There is a discussion on health improvements through water sanitation and hygiene as well as food security and nutrition. | Climate risks identified and illustrative actions and responses for all climate risks provided | Some CC risks to health mentioned, in relation to COVID-19. Health responses captured relate to mitigation such as BC actions to improve resource efficiency and building retrofitting. Links drought and health |
| 3^rd^ Edition Environmental Implementation Plan (2019) | Department of Environmental Affairs and Development Planning (DEADP) | Adaptation content is limited. One of the priority outcomes for DEADP is ‘responding effectively to Climate Change (mitigation and adaptation)’ of which mainstreaming of climate response was said to be achieved in 2018/2019. | Health adaptation is not discussed | No discussion of climate change risks. | No health information provided |
| WC DEADP Strategic Plan 2020 - 2025 (2020) | Department of Environmental Affairs and Development Planning (DEADP) | The importance of adaptation is acknowledged and focuses on ecosystem-based adaptation. A provincial climate challenge is resourcing and implementing effective adaptation. | Health adaptation is not discussed | Limited information on climate risks only that drought and heat are threats. | No Health information provided |
| WC Health Strategic Plan 2020-2025 (2020) | Western Cape Department of Health | Adaptation is not discussed. There is mention of active development of mitigation and adaptation strategies in the department. | Health adaptation is not discussed | No climate risks discussed except mention of drought, fires and disasters worsened by climate change | Limited health information as relates to climate change. No evidence provided. |
| Western Cape Annual Performance Plan 2021/2022 (2021) | Western Cape Department of Health | Adaptation is not discussed. | Health adaptation is not discussed. There is mention that Provincial department of health is attempting energy and water efficiency in the context of climate change and stable supply of medicine. | No discussion of climate change risks. | No health information provided as it relates to climate change. |
| Western Cape Climate Change Response Strategy (WCCRS) (2022) | Department of Environmental Affairs and Development Planning (DEADP) | Adaptation is seen as necessary to adapt to changes that are already locked into the global climate system as well as further changes from increased GHG emissions. For the Western Cape, adaptation is specific to water security, food security, coastal impacts, impacts on biodiversity and ecosystem services, and impacts on the most vulnerable communities. There is mention of the important role subnational governments play in adaptation and mitigation actions necessary to tackle climate change that is implemented at this level of governance. | Health adaptation is described as needing prevention, protection, and response measures in the health sector to help the health system adapt to climate change. | Climate risks identified and illustrative actions and responses for drought, sea level rise, wildfires and general 'disasters' included | Little mention of health and no health specific targets outlined. |

**Annex 2**

**Motivation for Integration of Climate Change into Document:**

This element looked at the underlying motivations in each document to discern what particular attention was given to the integration of climate change adaptation and indicate if a document was just making a mention of climate change to ‘check a box’. The motivation ‘to indicate to domestic stakeholders Climate Change is considered’ was added to the original driving factors list by *Ranabhat* because South African documents indicated such a critical motivation for integration. The Subcategories assessed and seen in the Table below:

Subcategories:

Observed Climate Change and its impacts

Projected/ Future impacts of CC

Compatibility with other policies/ Mainstreaming

Gained International support

To indicate to domestic stakeholders CC is considered

Ratings: Yes, Somewhat, Unsure, and No

**Table 1: Motivating Factors for Integrating Climate Change Adaptation**

| **Document Name** | **Observed Climate Change and its impacts** | **Projected/ Future impacts of CC** | **Compatibility with other policies/ Mainstreaming** | **Gained International support** | **To indicate to domestic stakeholders CC is considered** |
| --- | --- | --- | --- | --- | --- |
| **National Climate Change Response White Paper** | **Y** | **Y** | N | U | **Y** |
| **National Climate Change Adaptation Strategy** | **Y** | **Y** | **Y** | **Y** | **Y** |
| **National Heat Health Action Guideline** | **Y** | **Y** | **Y** | N | **Y** |
| **National Climate Change and Health Adaptation Plan 2020- 2024** | **Y** | **Y** | **Y** | U | **Y** |
| **Western Cape Climate Change Response Strategy** | **Y** | **Y** | **Y** | **Y** | **Y** |
| **Health Care 2030 Road to Wellness** | N | S | **Y** | N | N |

**Measures for Health Adaptation:**

To better understand what the extent to which health is considered in mitigating risk and vulnerabilities of the impacts of climate change in South Africa, the *Measures for Health Adaptation* looked for the existence of measures related to adaptations for health across the nine documents and whether these are explicit, informative, or non-existent. Informative but not explicit would include information on hypothetical measures, and potential outcomes, but this information is not measurable or written as indicators.

Ratings: Included and Explicit, Included Information but not Detailed and Not included

**Implementation Process:**

*Implementation Process* is comprised of three main categories: **Implementation Plan**, **Resources**, and **Monitoring and Evaluation** that further detail elements within each category.

Under the **Implementation Plan** there is:

Institutional Set-Up – Details of specific institutions/organizations role and ability to work on climate change adaptation

Allocation of Responsibilities – Specified tasks and responsibilities are assigned to specific institutions at varied levels of governance

Timeline – specific timeframes and dates are included for implementation of activities

Under **Resources** there is:

Financial – The mention or discussion of how activities/ responsibilities will be financed including a plan to collect funds and complete budgets

Human – The mention or discussion of how human capacity will be addressed to fulfil suggested activities/ responsibilities

Under **Monitoring and Evaluation** there is:

Framework and Reporting – a structured framework or discussion of reporting standards and structures is included

Lessons Learned – A discussion on importance of and how lessons learned will be incorporated in future documents and activities

Ratings: Included and Explicit, Included Information but not Detailed and Not included
